# Metformin turns off the metabolic switch of pancreatic cancer

**DOI:** 10.18632/aging.102622

**Published:** 2019-12-12

**Authors:** Xavier Deschênes-Simard, Marie-Camille Rowell, Gerardo Ferbeyre

**Affiliations:** 1 CR-CHUM, Université de Montréal, Montréal, Québec, Canada

**Keywords:** pancreatic cancer, cellular senescence, tumor-initiating cells, mitochondria, metformin

Oncogene-induced senescence (OIS) is a putative tumor-suppressor mechanism activated by oncogenic driver mutations. Cells with a senescent phenotype are mostly observed in premalignant lesions, including preinvasive pancreatic intraepithelial neoplasias (PanINs). Bypass of OIS in these lesions is a prerequisite to progression towards malignant cancer and is associated with a significant gene expression reprogramming. Our group was interested in comparing the gene expression profile of PanINs with pancreatic ductal adenocarcinoma (PDAC) cells, both harboring a driving mutation in the GTPase KRAS. We found significant changes regarding genes involved in differentiation, proliferation and metabolism [1].

**Figure 1:**
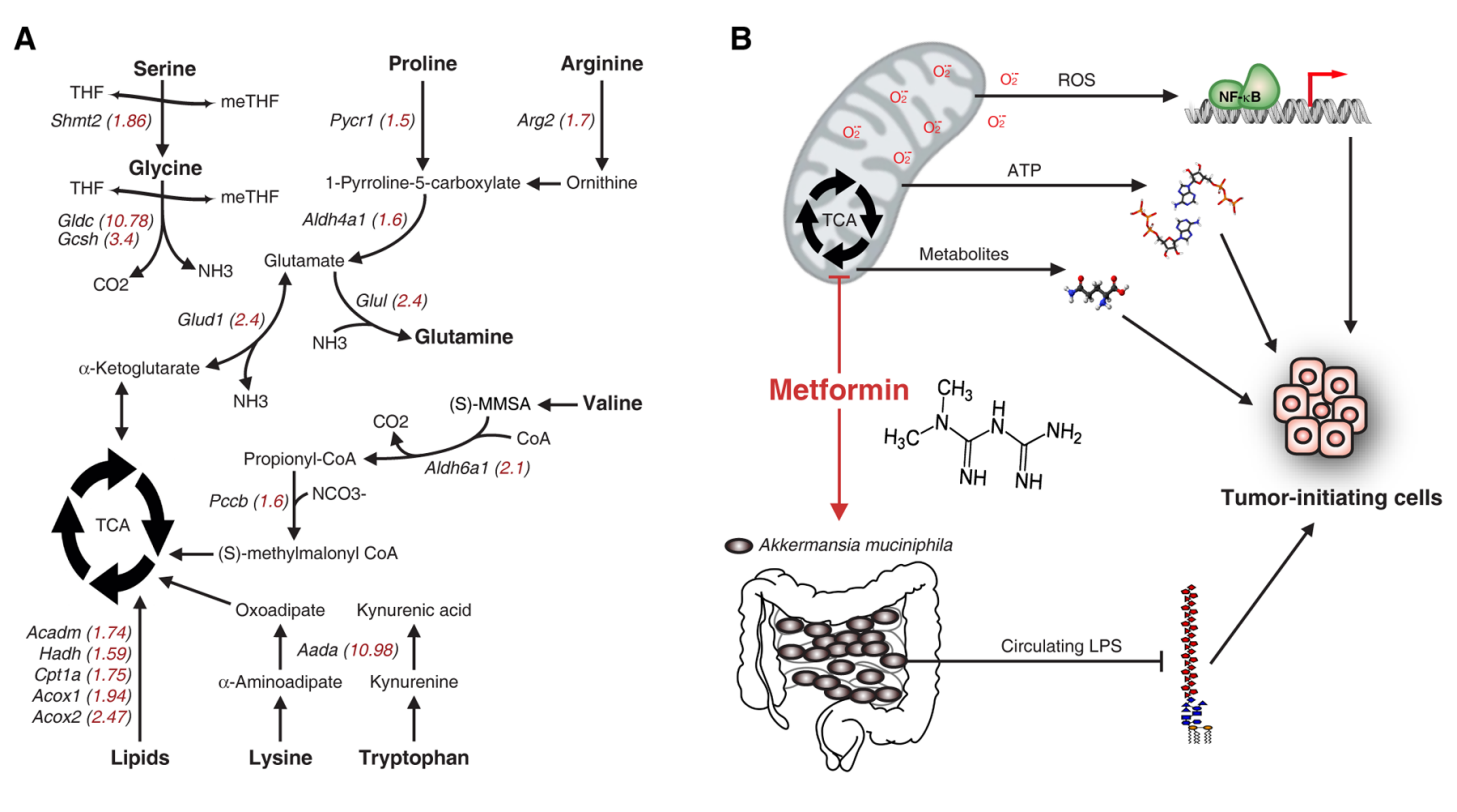
**Mitochondrial metabolism and possible mechanisms of metformin sensitivity in pancreatic tumor-initiating cells.** (**A**) Increased mitochondrial gene expression in pancreatic cancer cells compared to PanIN cells. An analysis by microarray was performed as described in Deschenes-Simard et al. and analysed with the FlexArray 1.6.3 software (GEO accession number: GSE57566). The fold increase of relative mRNA levels is indicated in red inside brackets. (**B**) Potential mechanisms explaining the specific toxicity of metformin on tumor-initiating cells. The inhibition of the TCA cycle by metformin may; impair ROS-dependent signaling, induce bioenergetic stress and reduce the production of critical metabolites required for tumor-initiating cell survival. In addition, metformin may increase the abundance of *Akkermansia muciniphila* in the gastrointestinal tract, thus protecting against the translocation of LPS in the systemic circulation.

A striking difference of the transcriptome of PDAC was an increased expression of genes involved in mitochondrial functions. Of these, many are involved in amino acid metabolism (Figure 1A). This observation was in keeping with an accumulating body of evidence proposing a critical role for mitochondrial biogenesis in pancreatic cancer progression [2]. We also observed that pancreatic cancer cells with a more dedifferentiated phenotype and capable of forming new tumors have a higher mitochondrial content, thereby suggesting a correlation between tumor-initiating cells and mitochondrial addiction. This hypothesis suggests that targeting mitochondria might be a valuable approach to kill tumor-initiating cells, which have been linked to resistance to several current therapies, tumor recurrence and metastatic tumor formation.

Metformin is a cheap antihyperglycemic drug from the biguanide family used for decades as a first line treatment for type 2 diabetes. Since the beginning of the twenty-first century, accumulating observational, experimental and clinical studies reported that this drug reduces cancer risk [3]. The exact mechanism of action to explain an anti-neoplastic effect of metformin and its derivatives remains to be fully elucidated. However, many studies are pointing to its capacity to modulate mitochondrial metabolism, at least partly by an inhibition of the mitochondrial respiratory-chain complex 1 [4]. Some groups have also demonstrated that metformin toxicity is specific for dedifferentiated cancer cells; i.e. cancer stem cells or tumor-initiating cells [2]. Our findings are consistent with the above observations. Indeed, we found that metformin exerts its toxicity towards cancer cells with a dedifferentiated phenotype and harbouring high mitochondrial content. This may be explained by a bioenergetic stress resulting from an inability of PDAC tumor-initiating cells to turn on glycolysis for ATP production upon inhibition of the oxidative phosphorylation (OXPHOS) by metformin [2]. This is contrary to other cancer cells forming the bulk of the tumor relying mainly on glycolysis for ATP production (Warburg effect) and senescent cells which can be highly glycolytic as well. Another hypothesis is an addiction on the tricarboxylic acid (TCA) cycle for production of critical macromolecules or amino-acid metabolism in tumor-initiating cells. With inactivation of complex 1 by metformin, the production of metabolites by the TCA cycle is compromised due to inhibition of key enzymes by increased levels of NADH. In an attempt to restore cellular ATP levels where compensatory activation of glycolysis cannot meet the cellular requirements, there is activation of AMPK and inactivation of mTORC1 in order to switch from anabolism to catabolism, thereby further compromising the synthesis of essential biomolecules [4]. A third hypothesis is that metformin inhibits signaling pathways induced by reactive oxygen species (ROS). For example, we have shown that metformin inhibits ROS production [5] and that this may explain a decreased activation of the NF-κB pathway [6]. A positive feedback loop between the NF-κB pathway, STAT3 and inflammatory mediators as IL6 has been shown to be required for the self-renewal of tumor-initiating cells [7].

Taken together, the hypotheses discussed above suggest that metformin could be a powerful pleotropic drug to eliminate tumor-initiating cells by acting at multiple levels (Figure 1B). Indeed, the mechanisms proposed above are likely not mutually exclusive according to the broad impact of complex 1 inhibition on cell metabolism. Also, the structural simplicity of metformin suggest that it may have other cellular targets, maybe contributing to other mechanisms of tumor suppression. Interestingly, emerging evidences suggest that metformin can have surprising effect on complex *in vivo* systems by modulating the microbiome. For example, metformin increases significantly the intestinal population of *Akkermansia muciniphila*, an anaerobic gram-negative bacterium [8]. This bacterium uses mucin as its source of carbon, and it is thought that mucin consumption helps to the turnover of mucus production and to reinforce the intestinal mucosal barrier. This is associated to reduced circulating lipopolysaccharide (LPS) and inflammation, thus maybe limiting the conversion of premalignant cells to tumor-initiating cells by LPS as our results suggest. In conclusion, much still to do to better characterize the potential role of metformin in cancer. However, resistance to therapies caused by tumor-initiating cells justify a clinical need to target these cells. The low cost of metformin, its availability and its favorable safety profile make this drug an interesting candidate.

PMID: 29018410
